# Non-coding RNA: A key regulator in the Glutathione-GPX4 pathway of ferroptosis

**DOI:** 10.1016/j.ncrna.2024.05.007

**Published:** 2024-05-20

**Authors:** Sadique Hussain, Gaurav Gupta, Moyad Shahwan, Pooja Bansal, Harpreet Kaur, Mahamedha Deorari, Kumud Pant, Haider Ali, Sachin Kumar Singh, Venkata Sita Rama Raju Allam, Keshav Raj Paudel, Kamal Dua, Vinoth Kumarasamy, Vetriselvan Subramaniyan

**Affiliations:** a Uttaranchal Institute of Pharmaceutical Sciences, Uttaranchal University, Dehradun, India; b Centre of Medical and Bio-allied Health Sciences Research, Ajman University, Ajman, 346, United Arab Emirates; c Department of Clinical Sciences, College of Pharmacy and Health Sciences, Ajman University, Ajman, 346, United Arab Emirates; d Department of Biotechnology and Genetics, Jain (Deemed-to-be) University, Bengaluru, Karnataka, 560069, India; e Department of Allied Healthcare and Sciences, Vivekananda Global University, Jaipur, Rajasthan, 303012, India; f School of Basic & Applied Sciences, Shobhit University, Gangoh, Uttar Pradesh, 247341, India; g Department of Health & Allied Sciences, Arka Jain University, Jamshedpur, Jharkhand, 831001, India; h Uttaranchal Institute of Pharmaceutical Sciences, Uttaranchal University, Dehradun, India; i Graphic Era (Deemed to be University), Clement Town, Dehradun, 248002, India; j Graphic Era Hill University, Clement Town, Dehradun, 248002, India; k Centre for Global Health Research, Saveetha Medical College, Saveetha Institute of Medical and Technical Sciences, Saveetha University, India; l Department of Pharmacology, Kyrgyz State Medical College, Bishkek, Kyrgyzstan; m School of Pharmaceutical Sciences, Lovely Professional University, Phagwara, Punjab, 144411, India; n Faculty of Health, Australian Research Centre in Complementary and Integrative Medicine, University of Technology Sydney, Ultimo, NSW, 2007, Australia; o Department of Medical Biochemistry and Microbiology, Uppsala University, Uppsala, Sweden; p Centre for Inflammation, Centenary Institute and University of Technology Sydney, School of Life Sciences, Faculty of Science, Sydney, NSW, 2007, Australia; q Discipline of Pharmacy, Graduate School of Health, University of Technology Sydney, P.O. Box: 123 Broadway, Ultimo, NSW, 2007, Australia; r Department of Parasitology and Medical Entomology, Faculty of Medicine, Universiti Kebangsaan Malaysia, Jalan Yaacob Latif, Cheras, 56000, Kuala Lumpur, Malaysia; s Pharmacology Unit, Jeffrey Cheah School of Medicine and Health Sciences, Monash University Malaysia, Jalan Lagoon Selatan, Bandar Sunway, 47500, Selangor Darul Ehsan, Malaysia; t School of Medical and Life Sciences, Sunway University, 47500 Sunway City, Malaysia; u Chitkara College of Pharmacy, Chitkara University, Rajpura 140401, Punjab, India

**Keywords:** Ferroptosis, ncRNAs, Cancer, GSH-GPX4, Cell death

## Abstract

Ferroptosis, a form of regulated cell death, has emerged as a crucial process in diverse pathophysiological states, encompassing cancer, neurodegenerative ailments, and ischemia-reperfusion injury. The glutathione (GSH)-dependent lipid peroxidation pathway, chiefly governed by glutathione peroxidase 4 (GPX4), assumes an essential part in driving ferroptosis. GPX4, as the principal orchestrator of ferroptosis, has garnered significant attention across cancer, cardiovascular, and neuroscience domains over the past decade. Noteworthy investigations have elucidated the indispensable functions of ferroptosis in numerous diseases, including tumorigenesis, wherein robust ferroptosis within cells can impede tumor advancement. Recent research has underscored the complex regulatory role of non-coding RNAs (ncRNAs) in regulating the GSH-GPX4 network, thus influencing cellular susceptibility to ferroptosis. This exhaustive review endeavors to probe into the multifaceted processes by which ncRNAs control the GSH-GPX4 network in ferroptosis. Specifically, we delve into the functions of miRNAs, lncRNAs, and circRNAs in regulating GPX4 expression and impacting cellular susceptibility to ferroptosis. Moreover, we discuss the clinical implications of dysregulated interactions between ncRNAs and GPX4 in several conditions, underscoring their capacity as viable targets for therapeutic intervention. Additionally, the review explores emerging strategies aimed at targeting ncRNAs to modulate the GSH-GPX4 pathway and manipulate ferroptosis for therapeutic advantage. A comprehensive understanding of these intricate regulatory networks furnishes insights into innovative therapeutic avenues for diseases associated with perturbed ferroptosis, thereby laying the groundwork for therapeutic interventions targeting ncRNAs in ferroptosis-related pathological conditions.

## 1 Introduction

**Fig. 1:**
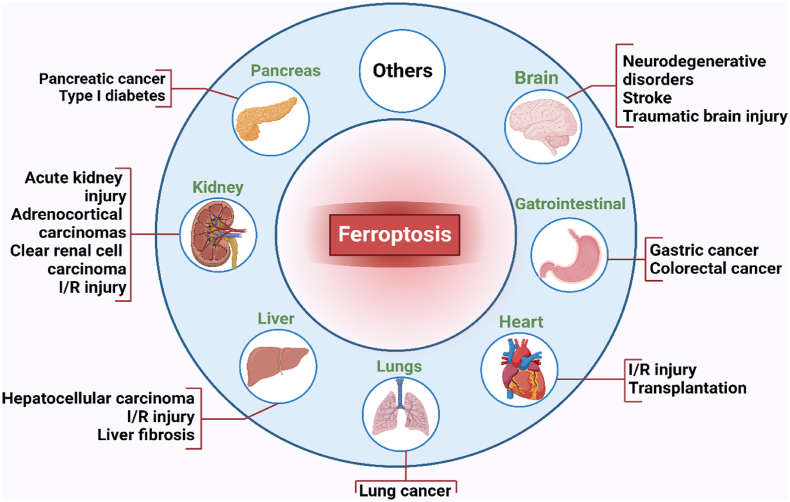
Ferroptosis has been associated with crucial functions in numerous pathological conditions.

Programmed cell death (PCD) holds significance in maintaining the equilibrium between disease progression and wellness [1]. Ferroptosis, coined as a new type of PCD in 2012, stands apart from other forms of cell death [2,3]. Numerous investigations have elucidated that ferroptosis represents a distinctive oxidative and iron-dependent PCD manifestation resulting from aberrant iron metabolism, consequential lethal lipid peroxidation (LPx), and Glutathione (GSH) depletion [4,5]. GSH is a peptide comprised of glutamate, cysteine, and glycine, serving a vital function in neutralizing reactive oxygen species (ROS) within cellular environments. In the realm of ferroptosis, the reduction of GSH levels emerges as a pivotal contributor to the initiation of this mode of cell death [6]. Furthermore, specific studies have underscored the critical involvement of autophagy in ferroptosis, particularly in the autophagic degradation of ferroptosis-related (Fr–R) proteins [7,8]. The involvement of ferroptosis in various diseases has gained increasing attention [[9], [10], [11]]. Ferroptosis is orchestrated through signal transduction routes including iron buildup, LPx, and cell membrane degradation. Notably, drugs or genetic interventions can modulate ferroptosis [12]. The primary mechanism of ferroptosis revolves around maintaining homeostasis between oxidative and antioxidant systems [13]. The loss of repair activity for lipid peroxides by glutathione peroxidase 4 (GPX4), the presence of iron that may undergo redox reactions, and the degradation of phospholipids which consist of polyunsaturated fatty acids (PUFA) serve as unique indicators [14]. Multiple processes, including amino acid (AA) and the breakdown of iron, cell adhesion, ferritinophagy, Keap1/Nrf2, p53, and phospholipid synthesizing, are recognized as modifiers of susceptibility to ferroptosis [[14], [15], [16], [17]]. Ferroptosis has a crucial regulatory function in a range of disorders, including carcinogenesis, ischemia-reperfusion damage, kidney damage, neurological ailments, and hemorrhagic conditions. Therapeutic approaches directed at the ferroptosis pathway and the metabolic vulnerabilities associated with ferroptosis have been documented [16,[18], [19], [20]]. Fig. 1 shows the association of ferroptosis with several pathological functions.

In 1982, researchers first isolated a novel GPX from pig liver [21]. Unlike previously identified GPX 1–3, this newly discovered enzyme, phospholipid hydroperoxide GPX (PHGPX), directly targets peroxidized phospholipids within cell membranes. While GPX1-3 functions as tetramers, primarily reducing H2O2 and fatty acid hydroperoxides [22,23], PHGPX operates as a monomeric protein, specifically reducing lipid hydroperoxides [22]. Subsequently, PHGPX underwent a name change to become known as GPX4 [23]. It was not until 2014 that GPX4 was recognized as the pivotal controller of ferroptosis [4]. The GPX4 network assumes a pivotal part in the modulation of ferroptosis. Various research has emphasized the importance of GPX4 in modulating ferroptosis and its implications across different conditions. Li et al. revealed that dexmedetomidine mitigates diabetic cardiomyopathy by blocking the process of ferroptosis via the Nrf2/GPX4 system, suggesting prospective possibilities for therapeutic intervention in this disease [24]. Activation of creatine kinase B (CKB) was identified to phosphorylate GPX4, preventing ferroptosis and fostering tumor growth in mice. The non-metabolic role of CKB enhances the durability of GPX4, unveiling an intricate process through which tumor cells encounter ferroptosis [25,26]. Investigations examining the impact of intense physical activity on the intestines as well as initial brain damage after subarachnoid hemorrhage revealed the preventive benefits of resveratrol and netrin-1, respectively, through the Nrf2/FTH1/GPX4 and PPARγ/Nrf2/GPX4 cascade [27,28]. Hemin was identified to alleviate contrast-induced nephropathy by suppressing ferroptosis via the HO-1/Nrf2/GPX4 cascade. In parallel, Dendrobium nobile polysaccharides were shown to attenuate ferroptosis and enhance cognitive abilities in rats with vascular dementia, underscoring the involvement of the GPX4 pathway in these contexts [29,30]. FOXA2 suppression by TRIM36 was found to exert an anti-cancer function in colorectal cancer (CRC) by promoting NRF2/GPX4-mediated ferroptosis, emphasizing the potential of targeting the Nrf2/GPX4 pathway for therapeutic interventions in cancer [31,32]. These collective findings underscore the GPX4 pathway's importance as a potential target for developing therapeutic strategies aimed at modulating ferroptosis across various pathological contexts.

Non-coding RNAs (ncRNAs) represent a diverse category of RNA molecules with limited protein-coding ability, playing essential cellular roles through various molecular mechanisms [33]. Initially perceived as functionally irrelevant, the emergence of numerous functional ncRNAs has substantially challenged the conventional notion of proteins as the exclusive functional products of gene expression [32,34]. In a broad classification, ncRNAs may be classified into two groups based on the length of their RNA transcripts: short and long [35]. Short ncRNAs typically refer to molecules with a length of approximately 200 nucleotides or less, including microRNAs (miRNAs), small interfering RNAs (siRNAs), and piwi-interacting RNAs (piRNAs). On the other hand, long ncRNAs (lncRNAs) are generally defined as transcripts longer than 200 nucleotides that lack protein-coding potential [36]. Three primary classes of ncRNAs include circular RNAs (circRNAs), lncRNAs, and miRNAs. With inherent characteristics that may exhibit tissue- and disease-specificity, these ncRNAs can be assessed in various body fluids, rendering them intriguing for possible use as indicators [37]. Furthermore, ncRNAs often undergo dysregulation in diverse illnesses, notably in cancer, with reported involvement in drug resistance (DR) in several instances [[38], [39], [40], [41], [42]]. Consequently, targeting ncRNAs holds promise as a therapeutic avenue to modulate DR-promoting pathways in cancer cells, presenting an opportunity to enhance patient outcomes [[43], [44], [45]].

The review aims to thoroughly assess and consolidate the latest knowledge regarding the intricate regulatory functions performed by ncRNAs within the GSH-GPX4 pathway of ferroptosis. This comprehensive review seeks to analyze existing research findings, delineating the influence of ncRNAs on the regulation of crucial constituents of the GSH-GPX4 pathway and their contribution to the modulation of ferroptosis. The review endeavors to furnish a thorough comprehension of the essential functions of ncRNAs in the GSH-GPX4 pathway of ferroptosis, providing valuable insights for researchers and clinicians involved in Fr–R investigations and therapeutic advancements.

## 2 Structure and function of GPX4 and GPX family

GPX4 plays a crucial role as an oxidoreductase within the GPX family, regulating ROS levels. The GPX4 protein, which contains selenium (Se), is the 4th gene of the GPX complex. The gene is situated at position 19p3.3 within the genome of a person and is composed of seven exons. These exons encode a sequence of peptides that is made up of 170 AA sequences. The projected molecular mass of this chain is 19 kDa [46,47]. GPX4 comprises a thioredoxin domain that is distinguished by four α-helices and seven β-strands that are subjected to the environment. GPX4 has three crucial residues inside its active sites: glutamine (Q81), selenocysteine (U46), and tryptophan (W136). These remains have a crucial significance in determining the function of GPX4 [42,48,49].

In mammals, there exist, various members of the GPX4 group, encompassing GPX1 to GPX8, which are categorized into three groups based on their similarities and differences in AA patterns: GPX1 to GPX3, GPX5 to GPX6, and GPX4 along with GPX7 to GPX8. GPX1 to GPX4 contain Se, while GPX6 incorporates selenocysteine (Sec), an essential active site [50]. Notably, GPX1 to GPX4 is capable of shielding against oxidative attacks and inflammation inhibition, although the specific function of GPX6 remains unclear [51]. GPX1 and GPX4 primarily curb the phosphate network by hindering the suppression of lipid hydrogen peroxide to phosphatase. While GPX2 controls the regenerative equilibrium of intestinal cells and suppresses inflammation-induced bowel cancer, it also fosters the proliferation of tumors. Conversely, GPX3 is recognized as a tumor suppressor. The remaining GPX family members feature cysteine as their active site. Recent research has highlighted a novel role for GPX4 and GPX5 in male fertility. Additionally, GPX7 interacts with GPX8 and protein isomerase, playing a distinct process of oxidation of protein within the endoplasmic reticulum [52,53]. Unlike its counterparts, GPX4 proficiently eliminates membrane lipid hydrogen peroxide products to counteract LPx for preserving lipid redox stability and thwarting ferroptosis [54].

GPX4 stands out as the sole enzyme within the GPX family, with the direct capability to reduce and neutralize lipid hydroperoxides. Unlike GPX5 and GPX7-GPX8, the active sites of GPX5 and GPX7-GPX8 lack Sec, instead featuring cysteine residues (Cys) as their active centers. GPX5 predominantly expresses itself in epididymal tissue, where it serves to shield sperm cells from oxidative damage. Both GPX7 and GPX8 are situated in the endoplasmic reticulum, fulfilling indispensable roles in mediating the oxidative folding process of proteins within this cellular organelle. GPX8, in particular, assumes a crucial role in facilitating the oxidative folding of endoplasmic reticulum proteins [55].

**Fig. 2:**
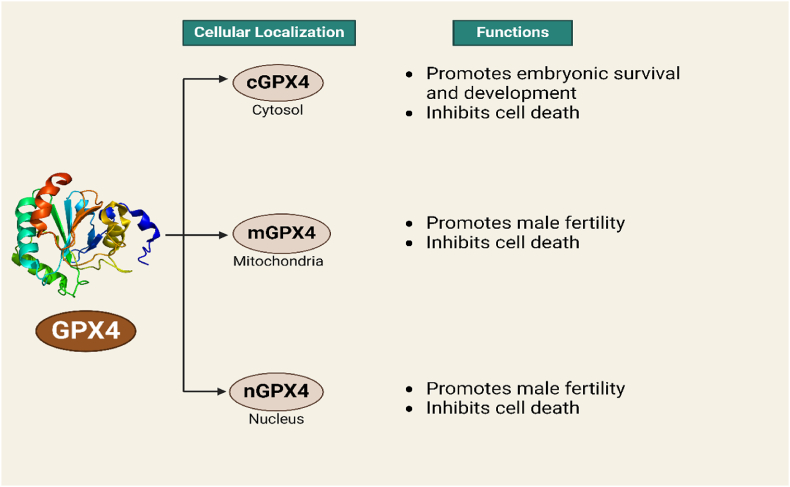
The three distinct isoforms of the GPX4 protein: cytosolic GPX4 (cGPX4), mitochondrial GPX4 (mGPX4), and nuclear GPX4 (nGPX4). Their subcellular localization and functional differences.

GPX4 demonstrates a diverse tissue distribution, with the highest concentration observed in the testes, where it significantly influences sperm development and function [56]. Physiologically, GPX4 exists in three distinct isoforms: cytosolic (cGPX4), mitochondrial (mGPX4), and nuclear (nGPX4), depicted in Fig. 2 [[57], [58], [59], [60], [61]]. In addition to its role in safeguarding against infertility, mGPX4 serves to mitigate cell death prompted by mitochondrial ROS or oxidized α-ketoisocaproic acid [62,63]. Notably, cGPX4 possesses the ability to translocate into the nucleus [64], whereas mGPX4 harbors a mitochondria-targeting sequence consisting of the N-terminal 27 AAs [65], and nGPX4 features a canonical nuclear localization signal alongside lysine/arginine-rich domains, akin to those present in protamines. These domains facilitate the binding of nGPX4 to sperm DNA, facilitating the oxidation of cysteines within protamines [66]. Deeper insights into the localization patterns and regulatory signals governing GPX4 variants hold promise for the creation of novel treatment strategies aimed at reducing oxidative harm.

## 3 Regulation of non-coding RNAs

Historically perceived as non-functional or “junk” patterns because of their lack of proficiency in protein coding, ncRNAs are now widely acknowledged for their crucial functions in diverse physiological processes, including gene modulation, cellular functioning, and the development of diseases [61,[67], [68], [69], [70]]. The ncRNAs may be generally classified into two primary classes: housekeeping and regulatory ncRNAs. Housekeeping ncRNAs include examples such as transfer RNAs (tRNAs), ribosomal RNAs (rRNAs), and small nuclear RNAs (snRNAs), which play essential roles in fundamental cellular processes such as translation, protein synthesis, and RNA splicing. They play a vital part in crucial tasks such as protein formation and splicing of pre-mRNA [71,72]. Regulatory ncRNAs have a significant function in controlling gene activity and other cellular functions. The examples include microRNAs (miRNAs), long non-coding RNAs (lncRNAs), and circular RNAs (circRNAs), which are known to exert regulatory functions by modulating gene expression at the transcriptional or post-transcriptional level [73]. NcRNAs have a pivotal function in the advancement of tumors, affecting several facets of tumor development. These ncRNAs have a vital function in directing immunological barriers, therefore impacting the growth, specialization, and general progression of tumors [74,75]. MiRNAs function as post-transcriptional regulators by linking specifically to the 3′ untranslated region (UTR) of targeted mRNAs. They can act as either cancer-promoting or inhibiting genes, depending on their target genes, and play roles in regulating cell angiogenesis, death, differentiation, and proliferation [76]. CircRNAs are closed loops formed by covalent bonds, exhibit immunity to exonucleases, and function as mimics for miRNAs. By conserving miRNAs, circRNAs mitigate their capacity to inhibit specific mRNAs. Specific circRNAs have been recognized as regulators or blockers of cancer advancement via the modulation of miRNA function [72,[77], [78], [79]]. LncRNAs have several functions in controlling gene expression. They interact with miRNAs as competitive endogenous RNAs (ceRNAs) to regulate the accessibility of miRNAs to their intended mRNAs. LncRNAs play a significant part in several biological activities, including the modification of chromatin structure, alterations in gene activity due to epigenetic factors, and regulation of gene expression [80,81]. The complex networks of regulation established by the associations of miRNAs, lncRNAs, and circRNAs have substantial consequences for several diseases [82,83]. The therapeutic relevance of ncRNAs in diseases is rapidly expanding, as they have an opportunity to serve as diagnostics, predictive, and treatment markers. They provide useful information about the many variants of the ailment and prospective targets for treatment [84]. The continuing investigation is revealing the complex mechanisms by which ncRNAs impact disease. These molecules show potential for developing new methods of detecting and managing diseases.

## 4 Non-coding RNA families involved in regulating the GSH-GPX4 pathway

### 4.1 miRNAs

#### 4.1.1 miRNAs targeting GSH synthesis

The cystine/glutamate antiporter (system xc−) consists of solute carrier family member 7A11 (SLC7A11) and SLC3A2. Its primary role involves facilitating the translocation of cystine across the membrane with glutamate [85,86]. Glutamic acid (GLA) is synthesized from glutamine degradation catalyzed by glutaminase (GLS, comprising GLS1 and GLS2), with GLS2 being a target of the cancer inhibitor gene P53 [[87], [88], [89]]. Thioredoxin reductase 1 (TXNRD1) reduces cystine exchange into cells to cysteine, which, along with GLA and glycine, undergoes two-step catalysis by GLA cysteine ligase (GCL) and GSH synthetase (GSS) to produce GSH [16,90]. Suppression of system xc− activity with elastin obstructs GSS, resulting in intracellular aggregation of lipid peroxide and subsequent induction of ferroptosis [91].

MiR-375 contributes to islet formation, release of insulin, and growth of cells [92]. It exhibits downregulation in gastric cancer (GC), where its upregulation significantly reduces the population of these stem cells. Mechanistically, miR-375 induces ferroptosis with particular emphasis on SLC7A11. In a xenograft model with GC cells implanted in nude mice, miR-375 suppresses tumor growth and diminishes stem cell frequency. Even when a limited quantity of cells are injected, miR-375 may suppress tumor formation in mice, and these effects are reversed upon overexpression of SLC7A11 [93]. Consequently, the miR-375/SLC7A11 cascade emerges as a suggested candidate for triggering ferroptosis in GC.

MiR-5096 exhibits inhibitory effects on breast cancer (BrC) metastasis while inducing cell death, potentially through ferroptosis induction. Its overexpression leads to elevated iron levels, ROS, hydroxyl radical, and lipid peroxide, accompanied by reduced GSH levels in BrC cells. This characteristic profile suggests its involvement in ferroptosis. Mechanistic investigations reveal that miR-5096 prompts ferroptosis in BrC cells by suppressing SLC7A11 function [94,95].

Lidocaine, a widely used local anesthetic in clinical settings, demonstrates inhibitory effects on the rapid multiplication, infiltration, and movement of ovarian and BrC cells, along with increased intracellular Fe2+ and ROS levels. Subsequent research reveals that lidocaine downregulates SLC7A11 expression by upregulating miR-382-5p. Suppressing miR-382-5p effectively counteracts lidocaine-mediated ferroptosis in ovarian and BrC. Hence, targeting miR-382-5p holds promise for promoting ferroptosis in infected cells [96]. Additionally, levobupivacaine, another local anesthetic, impedes the growth of GC cells by triggering ferroptosis via the miR-489-3p/SLC7A11 cascade [97,98].

Numerous miRNAs have been identified as modulators of cancerous cell ferroptosis by targeting SLC7A11. For instance, in thyroid cancer, the miR-545-3p/SLC7A11 cascade [99], in human oral squamous cell carcinoma, the miR-125b-5p/SLC7A11 cascade and miR-34c-3p/SLC7A11 cascade [100,101], in cervical cancer, the circEPSTI1/miR-375, miR-409-3p, miR-515-5p/SLC7A11 cascade [102], in hepatocellular carcinoma (HCC), the circ0097009/miR-1261/SLC7A11 cascade [103], in prostate cancer (PrC), the c-Myc/miR-25-3p/SLC7A11 cascade [104], and in renal cell carcinoma, the lncRNA SLC16A1-AS1/miR-143-3p/SLC7A11 cascade [105], all involve miRNAs targeting SLC7A11.

#### 4.1.2 miRNAs targeting GPX4

GPX4 functionality heavily relies on the presence of Sec, which constitutes the primary active site. Any mutations in this residue result in a drastic reduction, up to 90 %, in GPX4 activity [50]. Se acts as the precursor for Sec synthesis, crucial for forming the active site of GPX4, thereby exerting a substantial part in the initiation and progression of ferroptosis. The synthesis of Sec-tRNA, vital for Sec maturation, relies on isopentenyl pyrophosphate (IPP) generated through the mevalonate (MVA) network, explaining the observed downregulation of GPX4 expression and consequent ferroptosis induction upon MVA pathway inhibition by statins [106]. Furthermore, due to differences in their pKa values, cysteine cannot effectively substitute Sec's role [107]. Sec predominantly exists in an ionic state at physiological pH, essential for its catalytic activity. The catalytic cycle of GPX4 involves two phases: initially, the active site's Sec residue reduces lipid peroxides to non-toxic phospholipid alcohols while itself becoming oxidized. In the subsequent phase, the oxidized Sec residues regain functioning through a decline in two molecules of GSH, leading to GSH oxidation to GSSG [48]. Interruption of GSH formation or excessive GSH consumption prevents the recovery of the GPX4 active site, hindering GPX4 activity and resulting in cell ferroptosis [108]. Moreover, the application of GPX4 suppressors directly hampers GPX4 function, impeding effective clearance of lipid peroxides and eventually resulting in cellular ferroptosis [75,109].

MiR-15a-3p exerts a pivotal regulatory function in several types of cancer, including CRC, where it also serves in ferroptosis. It has been observed to directly attach to the 3′-UTR of GPX4, inhibiting its activity and consequently leading to elevated intracellular levels of ROS, Fe2+, and malondialdehyde (MDA) [110]. Furthermore, miR-539 exhibits low expression levels in CRC, while TNF-α Induced protein 8 (TNFAIP8/TIP8) is overexpressed in this cancer type. TIP8 promotes CRC proliferation, migration, and angiogenesis. Mechanistically, miR-539 regulates TIP expression and indirectly downregulates GPX4 by stimulating the SAPK/JNK signaling, thereby inducing ferroptosis and suppressing CRC progression [111]. Hence, addressing miR-15a-3p and miR-539 is a potentially effective method to trigger ferroptosis in CRC cells.

MiR-324-3p exhibits significant downregulation in cisplatin-tolerant A549 lung adenocarcinoma (LAC) cells. Its overexpression reverses the cells' cisplatin resistance. MiR-324-3p directly targets and suppresses GPX4 expression. Conversely, the upregulation of GPX4 counteracts the cisplatin-sensitizing influence of miR-324-3p on LAC cells. Notably, the GPX4 suppressor RSL3 mimics the effects of miR-324-3p upregulation in LAC cells. Therefore, the miR-324-3p/GPX4 cascade represents a promising target for enhancing cisplatin sensitivity in LAC cells [112].

Metformin, a widely used hypoglycemic medication in clinical practice, has garnered attention for its anticancer properties [113]. In a study focusing on MDA-MB-231 BrC cells, metformin was found to elevate miR-324-3p expression, leading to ferroptosis. MiR-324-3p was identified as directly linked to the 3′-UTR of GPX4, resulting in GPX4 downregulation and subsequent cell ferroptosis [114]. Ketamine, an intravenous anesthetic, demonstrated inhibitory effects on liver cancer (LC) cell proliferation both in vivo and in vitro, inducing ferroptosis. This effect was associated with decreased expression of lncPVT1 and GPX4. Further investigations revealed that lncPVT1 could interact directly with miR-214-3p, impeding its binding to GPX4. Silencing of lncPVT1 induced ferroptosis, while inhibition of miR-214-3p or overexpression of GPX4 reversed this process. Ketamine-induced ferroptosis could also be reversed by suppressing miR-214-3p or overexpressing GPX4. Therefore, ketamine modulates LC cell ferroptosis through the lncPVT1/miR-214-3p/GPX4 cascade [115].

The miR-1287-5p/GPX4 cascade regulates the proliferation and ferroptosis of human osteosarcoma (OS), influencing their sensitivity to cisplatin [116]. Similarly, in papillary thyroid cancer, the circKIF4A/miR-1231/GPX4 cascade [117], in PrC, the miR-15a/GPX4 cascade [118], in HCC, the circIL4R/miR-541-3p/GPX4 cascade [119], and in non-small cell lung cancer (NSCLC), the circDTL/miR-1287-5p/GPX4 cascade [120], all regulate the ferroptosis process in their respective tumor types.

#### 4.1.3 miRNAs modulate the GSH-GPX4 system

**Fig. 3:**
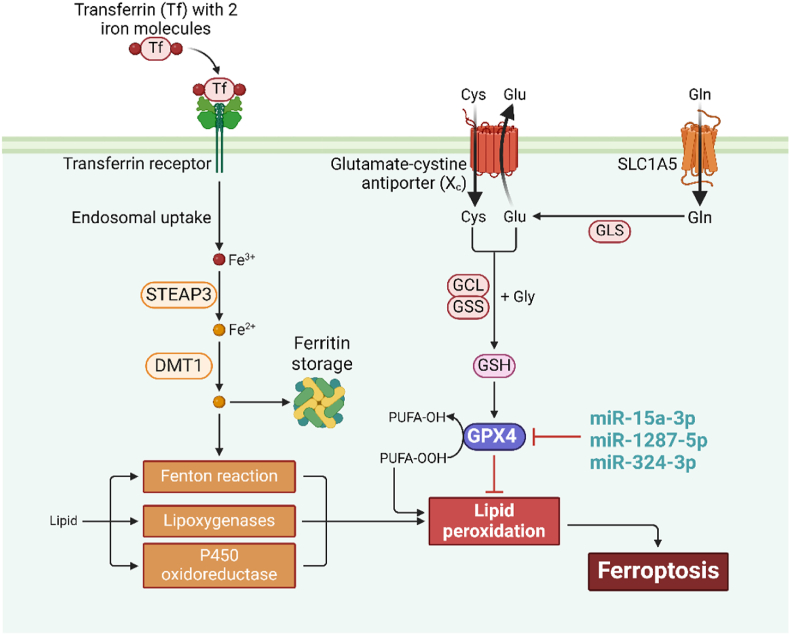
The processes through which microRNAs (miRNAs) regulate ferroptosis by targeting GPX4. miRNAs potentially exert their functions by directly binding to GPX4.

Recent findings indicate that miRNAs serve a crucial part in modulating many essential stages of ferroptosis, which include the GSH-GPX4 system, system xc− transportation, iron metabolism, and lipid metabolism. GPX4 operates as a GSH-dependent enzyme responsible for converting harmful lipid hydroperoxides into harmless lipoalcohol (L-OH), thereby preventing the transformation of iron-induced lipid hydroperoxides into highly reactive lipid alkoxyl radicals, consequently inhibiting ferroptosis. Blocking GPX4 activity accelerates ferroptosis progression. For instance, miR-15 can suppress GPX4 expression by binding to the 3′-UTR of GPX4 mRNA [118], impeding the conversion of GSH to L-GSH Oxidized (GSSG), impeding the conversion of dangerous lipid peroxides into non-toxic-L–OH–,-escalating GSH and ROS levels as well as MDA concentrations, thereby hastening ferroptosis. Similarly, miR-15a-3p [110], miR-1287-5p [116], and miR-324-3p exert analogous effects in cancer by directly targeting GPX4 to positively modulate ferroptosis (Table 1 & Fig. 3) [112].

**Table 1:** miRNAs regulating Glutathione-GPX4 pathway.

miRNAs	Action on GPX4	Ferroptosis	Disease	References
miR-15a-3p	↓	+	Colorectal cancer	[101]
miR-1287-5p	↓	+	Osteosarcoma	[107]
miR-324-3p	↓	+	Lung adenocarcinoma	[103]

↓, Downregulation; +, Promote.

Moreover, activating transcription factor 4 (ATF4) belongs to the CREB/ATF family and functions both as a transcriptional activator and repressor during ferroptosis. MicroRNA-214-3p contributes to HCC carcinogenesis regulation, and inhibiting miR-214 directly enhances ATF4 expression [121]. ATF4 binds to an AA response element within the promoter region of SLC7A11, thereby boosting SLC7A11 transcription. Furthermore, ATF4, a crucial component of endoplasmic reticulum stress implicated in ferroptosis, upregulates heat shock 70 kDa protein 5 (HSPA5) expression via the PERK-ATF4-CHOP network, enhances GPX4 expression, suppresses ROS production, and inhibits ferroptosis [122].

### 4.2 LncRNAs targeting GPX4

LncRNAs exhibit diverse mechanisms to target GPX4. For instance, lncRNA MALAT1 has been demonstrated to regulate oxidative damage and cardiovascular diseases (CVDs) development by inducing GPX4 expression through the Nrf2 pathway, resulting in reduced production of ROS and inhibition of vascular smooth muscle cell (VSMC) development and movement [123]. Another illustration is provided by lncRNA NEAT1, which confers docetaxel resistance in PrC cells by suppressing miRNA-34a-5p and miRNA-204-5p, consequently increasing ACSL4 expression and promoting docetaxel tolerance [124]. Moreover, in-MRGPRF-6:1 enhances ox-LDL-induced macrophage ferroptosis by repressing GPX4 [125]. Additionally, lncRNA MEG3 inhibits elastin-induced ferroptosis in chondrocytes by modulating the miR-885-5p/SLC7A11 cascade [126]. In essence, lncRNAs exert their influence on GPX4 through interactions with various molecules, such as miRNAs and transcription factors, thereby regulating gene expression and cellular processes.

In their study, Bai & Tang (2021) illustrated that H19 exhibited decreased expression in cases of spontaneous abortion. They also observed a positive correlation between H19 expression and levels of Bcl-2 and GPX4 while noting a negative correlation with Bax expression. Furthermore, silencing H19 resulted in the downregulation of Bcl-2 and GPX4, along with an upregulation of Bax, both at the mRNA and protein levels [127]. He et al. (2021) investigate the effects of the anesthetic ketamine on LC cells, focusing on its impact on cell proliferation and ferroptosis. The research demonstrates that ketamine effectively suppresses the viability and proliferation of LC cells in both in vitro and in vivo settings while also stimulating ferroptosis. These effects are associated with decreased expression of lncPVT1 and GPX4. Further analysis reveals that lncPVT1 interacts directly with miR-214-3p, inhibiting its function as a sponge for GPX4. Depletion of lncPVT1 accelerates ferroptosis in LC cells, whereas inhibition of miR-214-3p and overexpression of GPX4 reverse this effect. The study concludes that ketamine-induced cell growth suppression and ferroptosis are mediated through the lncPVT1/miR-214-3p/GPX4 cascade [115]. Wu & Liu (2021) aimed to investigate the mechanisms underlying ferroptosis sensitivity in NSCLC, focusing on the role of NEAT1 and acyl-CoA synthetase long-chain family member 4 (ACSL4). NEAT1 was found to regulate the expression of ACSL4 and proteins associated with both ferroptosis and classical apoptosis pathways. Specifically, NEAT1 silencing combined with erastin treatment resulted in a greater decrease in ACSL4, SLC7A11, and GPX4 levels compared to erastin treatment alone. NEAT1 was identified as a regulator of ferroptosis and ferroptosis sensitivity in NSCLC, with ACSL4 performing a crucial function in this procedure. Targeting NEAT1 or ACSL4 may offer a promising therapeutic strategy for NSCLC by enhancing ferroptosis sensitivity [128].

**Fig. 4:**
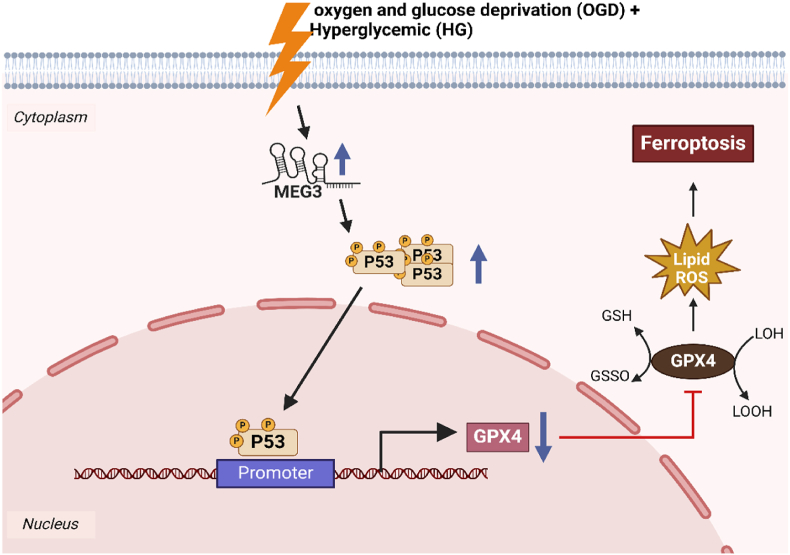
The representation illustrates the Meg3-p53-GPX4 pathway that regulates ferroptosis induced by OGD + HG damage. The combination of OGD and hyperglycemic reperfusion resulted in an increase in Meg3 activity. Furthermore, there was an association seen between Meg3 and p53 expression in RBMVECs. Afterwards, p53 suppressed the function of GPX4 by attaching to its activator. The knockdown of GPX4 resulted in the buildup of lipid peroxides, ultimately leading to the process of ferroptosis. Furthermore, the reduction of Meg3 was beneficial in safeguarding RBMVECs from ferroptosis generated by OGD + HG.

Diabetic individuals are at an increased risk of experiencing a stroke during the perioperative period, mainly because of ongoing high blood sugar levels. The therapeutic and regulatory functions of MEG3 in diabetic brain ischemia damage have not been well understood. However, it appears to be associated with the regulation of ischemic stroke. Chen et al. (2021) discovered that manipulating the levels of p53, either by increasing or decreasing its expression, had a substantial impact on the activity of GPX4 in rat brain microvascular endothelial cells (RBMVECs) when subjected to a combination of oxygen and glucose deprivation (OGD) and high blood sugar levels therapy. The restoration of p53 into MEG3-knockdown cells resulted in the suppression of GPX4 activity. Chromatin immunoprecipitation tests provided evidence that p53 was specifically attached to the GPX4 regulator, indicating its role in modulating GPX4 transcription and expression (Fig. 4). Overall, these findings suggest that the MEG3-p53 signaling cascade mediates ferroptosis in RBMVECs following injury induced by OGD combined with hyperglycemic reperfusion by regulating GPX4 transcription and expression [129]. Lei et al. (2022) conducted a study to elucidate the function and underlying molecular mechanism of linc00976 in cholangiocarcinoma (CCA). They noted a significant upregulation of linc00976 in CCA, which correlated positively with unfavorable clinical characteristics. Furthermore, they found that linc00976 facilitated the proliferation and mobility of CCA cells while suppressing ferroptosis through modulation of the miR-3202/GPX4 cascade [130]. Kang et al. (2022) discovered that LINC01134 functions as a newly identified suppressor of ferroptosis, elevating GPX4 levels by facilitating the recruitment of the transcription factor Nrf2 to the GPX4 promoter. Consequently, this mechanism enhances LC's resistance to Oxaliplatin (OXA). They identified the LINC01134/Nrf2/GPX4 cascade as a pivotal and novel pathway governing the growth and advancement of HCC. Manipulating the expression of GPX4, LINC01134, or Nrf2 could represent potential therapeutic avenues to overcome OXA resistance in HCC [131].

Endometriosis (EMs) is a prevalent condition among women of reproductive age, characterized by the growth of endometrial tissues outside the uterus. The abnormal proliferative and migratory capabilities of endometrial stromal cells (ESCs) contribute significantly to the progression of EMs. LncRNAs have emerged as key regulators in EM development and progression. One such lncRNA, ADAMTS9-AS1, is found to be aberrantly expressed in ectopic endometrium (ECM). This study aimed to elucidate the biological role of ADAMTS9-AS1 in ESC proliferation and migration and explore the underlying mechanism. Wan et al. (2022) discovered that ADAMTS9-AS1 expression exhibited a significant increase in patients with EM and a murine model of EM. Functionally, silencing ADAMTS9-AS1 in ESCs resulted in reduced cell viability and migration. Notably, inhibition of ADAMTS9-AS1 reduced ESC viability, an effect that was notably reversed by ferrostatin-1 (Fer-1), an inhibitor of ferroptosis, indicating ADAMTS9-AS1's involvement in ferroptosis regulation. Further mechanistic investigations unveiled ADAMTS9-AS1's role as a ceRNA by sequestering miR-6516-5p, consequently upregulating the expression of GPX4. This dysregulation led to heightened levels of ROS and MDA, along with decreased GPX4 expression following ADAMTS9-AS1 inhibition. These findings underscore the role of upregulated ADAMTS9-AS1 in promoting ESC proliferation and migration through modulation of the miR-6516-5p/GPX4-dependent ferroptosis network [132].

You et al. (2023) elucidated that lnc-MRGPRF-6:1 facilitates oxidized-low density lipoprotein (ox-LDL)-induced macrophage ferroptosis in coronary atherosclerotic disease (CAD) by suppressing GPX4 [125]. Zhang et al. documented that heightened expression of OTUD6B-AS1 led to the stabilization of TRIM16 by interacting with HuR, consequently augmenting GPX4-mediated ferroptosis and thereby mitigating radioresistance in CRC [133]. TMEM44-AS1 can elevate the expression levels of GPX4. Yang et al. performed an array of tests in a controlled environment. They found that increasing the levels of GPX4 might counteract the effects of TMEM44-AS1 elimination, hence promoting the growth, movement, and penetration of esophageal squamous cell carcinoma (ESCC) cells via ferroptosis. However, more research is necessary to clarify the process by which TMEM44-AS1 boosts GPX4 stabilization of mRNA via its association with IGF2BP2. The results suggest that the TMEM44-AS1-IGF2BP2-GPX4 cascade plays a role in the development and progression of ESCC, providing new diagnostic indicators and possible treatment options for individuals with ESCC [134].

### 4.3 CircRNAs targeting GPX4

CircRNAs play significant roles in the modulation of ferroptosis through the control of GPX4 expression. In GC, CircRHOT1 functions to impede ferroptosis in cancer cells by epigenetically governing GPX4 expression. CircRHOT1 exhibited elevated expression levels in GC tumor tissues compared to adjacent non-tumor tissues. Its overexpression in GC cells hindered ferroptosis by recruiting KAT5 to epigenetically enhance the expression and activity of GPX4 [135]. Furthermore, Circ0060467 acts as a sponge for miR-6805, promoting the progression of HCC by regulating the expression of AIFM2 and GPX4. By competing with AIFM2 and GPX4, Circ0060467 suppresses cancer cell ferroptosis through its interaction with miR-6805, thereby facilitating HCC progression [136]. Moreover, CircIL4R contributes to tumorigenesis and impedes ferroptosis in HCC by modulating the miR‐541‐3p/GPX4 cascade [119].

Xu et al. (2020) revealed that circIL4R functions as an oncogene to accelerate tumor development and suppress ferroptosis through the miR-541-3p/GPX4 cascade in HCC, unveiling a distinctive regulatory pathway circIL4R/miR-541-3p/GPX4 [119]. Chen et al. (2021) validated the involvement of the circKIF4A-miR-1231-GPX4 axis in papillary thyroid cancer. This axis functions as a ceRNA, exerting suppressive effects on cancer progression and metastasis [117]. Jin et al. (2022) documented that silencing of mmu_circRNA_0000309 contributes to DR against germacrone in diabetic nephropathy (DN) mice. mmu_circRNA_0000309 acts as a sponge for miR-188-3p, leading to the upregulation of GPX4 expression. This process results in the deactivation of ferroptosis-dependent mitochondrial function and inhibition of podocyte apoptosis [137]. Liu et al. (2022) investigated the function of circACAP2 in cervical cancer and found that circACAP2 modulated cellular ferroptosis via the miR-193a-5p/GPX4 network [138].

Jiang et al. (2023) sought to investigate the possible involvement and mechanism of circHIPK3 in gestational diabetes mellitus (GDM). They proposed that circHIPK3 could potentially enhance ferroptosis by modulating miR-1278/DNMT1 to influence GPX4 DNA methylation in HTR-8/SVneo cells cultured under high glucose conditions [139]. Liu and Li (2023) investigated the impact of circ_0016142 downregulation on HCC cell proliferation and found that it inhibited cell growth by promoting ferroptosis through modulation of the miR-188-3p/GPX4 pathway [140]. Li et al. (2023) examined the increased expression of circBLNK in OS tissues and cells, correlating with unfavorable patient prognosis. They elucidated the mechanism wherein circBLNK acts as a ceRNA to sequester miR-188-3p and enhance GPX4 expression, consequently driving OS tumorigenesis [141]. Table 2 enlists the ncRNAs involved in regulating the GSH-GPX4 pathway.

**Table 2:** ncRNAs involved in regulating the GSH-GPX4 pathway.

ncRNAs	Diseases	References
Circular RNAs		
CircRHOT1	Gastric cancer	[128]
Circ0060467	Hepatocellular carcinoma	[129]
CircIL4R	Hepatocellular carcinoma	[112]
circACAP2	Cervical cancer	[131]
mmu_circRNA_0000309	Diabetic nephropathy	[130]
circHIPK3	Gestational diabetes mellitus	[132]
circ_0016142	Hepatocellular carcinoma	[133]
circBLNK	osteosarcoma	[134]
Long non-coding RNAs		
MALAT1	Cardiovascular diseases	[116]
NEAT1	Non-small cell lung cancer	[121]
lnc-MRGPRF-6:1	Coronary atherosclerotic disease	[118]
H19	abortion	[120]
lncPVT1	Lung cancer	[108]
MEG3	diabetic brain ischemia	[122]
linc00976	cholangiocarcinoma	[123]
LINC01134	Hepatocellular carcinoma	[124]
OTUD6B-AS1	Colorectal cancer	[126]
ADAMTS9-AS1	Ectopic endometrium	[125]
TMEM44-AS1	Esophageal squamous cell carcinoma	[127]
Micro RNAs		
miR-15a-3p	Colorectal cancer	[103]
miR-1287-5p	Osteosarcoma	[109]
miR-324-3p	Lung adenocarcinoma	[105]
miR-539	Colorectal cancer	[104]

## 5 Therapeutic approaches for ncRNAs targeting ferroptosis

Ferroptosis holds significant relevance in cancer, neurodegenerative disorders (NDs), and CVDs. Investigations have linked Fr–R regulators to glioma patient survival, with evidence indicating that stimulating ferroptosis could enhance glioma sensitivity to chemotherapy drugs, indicating its possibility as a viable approach for treating glioma [142]. In NDs and strokes, emerging data underscore the critical involvement of ferroptosis in pathophysiological processes. Pharmacological intervention targeting ferroptosis using bioactive small-molecule compounds, known as ferroptosis inhibitors, represents a promising approach for managing these conditions [143,144]. The targeting of ferroptosis holds significant promise in the context of CRC therapy, where DR poses a substantial challenge [145]. Ferroptosis-based therapeutic strategies offer a renewed sense of optimism for CRC patients, highlighting the potential of ferroptosis modulation as a valuable approach in CRC management [146].

Nanotechnology presents a viable strategy for directing ferroptosis in the treatment of cancer through the targeted delivery of ferroptosis-inducing agents to malignant cells. Ferroptosis has been evidenced to impede tumor proliferation and revert tumor advancement across a spectrum of cancers, including LC, lung, renal, CRC, pancreatic, and ovarian malignancies [147]. An illustration of this is the application of nanoparticles to deliver antisense oligonucleotides (ASOs) in a targeted manner, aiming to regulate the expression of GPX4 within VSMCs. In a recent investigation by Liao et al., they discovered that ASO-MALAT1, designed to target the MALAT1, could suppress AngII-induced ROS generation, as well as the proliferation and migration of VSMCs, by enhancing the expression of GPX4. This effect was reversible with siRNA-GPX4. Additionally, GPX4 overexpression exhibited inhibitory effects on AngII-induced VSMC proliferation and migration. The mechanism underlying MALAT1 knockdown-induced GPX4 expression involves Nrf2 [148,149]. Another study conducted by Li et al. showcased the therapeutic potential of Shaoyao Decoction in ameliorating colitis through the regulation of GPX4-mediated ferroptosis in epithelial cells. Their findings demonstrated that Shaoyao Decoction could mitigate colitis by modulating the GPX4-regulated ferroptosis pathway within epithelial cells. This study underscores the promise of employing nanotechnology-driven delivery systems to target GPX4 and regulate the glutathione-GPX4 pathway for the management of colitis [150]. Tailored nanoparticles can be engineered to encapsulate and transport ferroptosis-triggering compounds to cancerous cells, augmenting treatment specificity and efficacy. For instance, a self-powered photodynamic therapeutic tablet has been innovated to amalgamate a ferroptosis inducer with photodynamic therapy, culminating in amplified ferroptosis within cancerous cells and notable tumor suppression. This innovative approach surmounts the hurdles associated with inadequate tissue penetration and tumor hypoxia inherent in conventional therapies while mitigating adverse effects [151]. Consequently, nanotechnology emerges as a potential avenue for the precision delivery of ferroptosis inducers in tumor management, potentially elevating therapeutic outcomes and circumventing DR [145,152]. The integration of therapeutics based on ncRNAs with nanotechnology holds promise for generating synergistic effects across diverse diseases and conditions, spanning cancer, NDs, CVDs, etc. This strategy has the potential to optimize treatment efficacy through enhanced drug delivery, precise targeting of molecular pathways, and mitigation of side effects.

## 6 ncRNAs-interaction among ferroptosis and other PCDs in cancer

Various forms of PCD, including apoptosis, autophagy, ferroptosis, and necroptosis, intricately interact with each other. This interplay is notably evident in the crosstalk between apoptosis and autophagy, apoptosis and necroptosis, and necroptosis and ferroptosis. ROS-induced LPx emerges as a pivotal mediator in mediating communication among these PCD pathways. LPx products, resulting from ROS-induced damage, exert deleterious effects by compromising DNA integrity, disrupting protein structure, and impairing enzymatic function across diverse cell types, ultimately impeding PCD. For instance, these LPx byproducts can trigger cell apoptosis via activation of signaling pathways such as NF-κB, MAPK, and protein kinase C-related systems. Furthermore, LPx can interfere with autophagy by modulating upstream modulators of autophagy-mediated systems, including AMP-activated protein kinase and Akt-mTOR cascade [153]. Moreover, the functioning of GPX4 plays an important part in regulating the extent of LPx and thereby influencing the induction of ferroptosis.

Numerous ncRNAs have been identified to modulate PCD, affecting the malignant advancement of tumors. The information presented illustrates the connection and parallels between ferroptosis and various types of cell demise. Undoubtedly, ncRNAs contribute to governing the interplay among these PCDs. This section offers a concise overview of pertinent research findings (Table 3).

**Table 3:** The involvement of ncRNAs in the interaction between ferroptosis and other mechanisms of cell death in malignancies.

NcRNAs	Role	Cancer	References
CircRNAs			
CircABCB10	↓ Apoptosis ↓ Ferroptosis	Rectal	[148]
Circ clARS	↑ Autophagy to facilitate ferroptosis	Liver	[145]
circDTL	↓ Apoptosis ↓ Ferroptosis	NSCLC	[111]
circLMO1	↑ Apoptosis ↑ Ferroptosis	Cervical	[149]
circRHOT1	↓ Apoptosis ↓ Ferroptosis	Breast	[150]
circ_0007142	↓ Apoptosis ↓ Ferroptosis	Colorectal	[151]
Circ_0000745	↓ Apoptosis ↓ Ferroptosis	ALL	[152]
LncRNAs			
H19	↓ Autophagy to facilitate ferroptosis	Breast	[153]
HCG18	↓ Apoptosis ↓ Ferroptosis	Liver	[154]
LINC01564	↓ Apoptosis ↓ Ferroptosis	Glioma	[155]
LINC00551	↑ Ferroptosis via autophagy-dependent mechanisms	Lung	[146]
LINC00618	↑ Ferroptosis via autophagy-dependent mechanisms	Leukemia	[156]
NEAT1	↓ Autophagy ↓ Ferroptosis	Melanoma	[147]
NEAT1	↑ Apoptosis ↑ Ferroptosis	Liver	[157]
OIP5-AS1	↓ Apoptosis ↓ Ferroptosis	Prostate	[158]
P53RRA (LINC00472)	↑ Apoptosis ↑ Ferroptosis	Lung	[143]
TMEM161B-AS1	↓ Apoptosis ↓ Ferroptosis	Glioma	[159]

↓, Inhibit; ↑, Promote.

Mao and his coworkers discovered that LINC00618 increases programmed cell death by raising the amounts of BCL2-related X (BAX) and cleavage caspase-3 while simultaneously inhibiting the transcription of SLC7A11 via lymphatic-specific decapping enzymes (LSH), which ultimately promotes ferroptosis. Importantly, the occurrence of ferroptosis caused by LINC00618 relies on vincristine (VCR)-induced apoptosis, highlighting its involvement in generating ferroptosis that is reliant on apoptosis. Furthermore, a multitude of ncRNAs have a role in the advancement of malignancies by concurrently controlling apoptosis and ferroptosis [154]. An instance of this is the lncRNA P53RRA, which has undergone methylation modification and is shown to be reduced in lung cancer. This reduction enables the movement of p53 into the nucleus by engaging with G3BP1. As a result, cell cycle stoppage, apoptosis, and ferroptosis occur [154,155]. Similarly, another research demonstrated that the cancer-causing component circDTL increases the expression of GPX4 by capturing miR-1287-5p, hence preventing ferroptosis and apoptosis [120].

The relationship between autophagy and ferroptosis seems to be close and interconnected. ALKBH5 functions as a suppressor of autophagic transition, whereas cIARS hinders ferroptosis by suppressing ALKBH5-induced autophagy, thereby increasing the susceptibility to sorafenib (SF) in HCC cells [156]. Furthermore, in LAC, the presence of LINC00551 limits the capacity of cells to survive by suppressing mTOR expression via the miR-4/DDIT4 cycle. This, in turn, increases the degree of autophagy and promotes ferroptosis in a way that relies on autophagy [157]. The latest research has shown that lincRNA NEAT1 has a role in the process of ferroptosis and autophagy triggered by gambogenic acid (GNA), a natural substance used in cancer treatment. This is achieved by regulating the SLC7A11/GPX4 and AMPK/mTOR pathways in melanoma [158]. There is a lack of reports elucidating the regulation of ncRNAs in cancerous cells regarding the interplay between ferroptosis and other PCD, necessitating further investigation into the corresponding regulatory relationships.

## 7 Clinical implications of ncRNAs and the GSH-GPX4 pathway

The relationship between ncRNAs and the GSH-GPX4 pathway has been explored across various contexts. For instance, in the realm of cancer stemness, m6A modification, a form of RNA alteration, can regulate the expression and functions of ncRNAs, thereby influencing cancer stemness properties. The precise mechanisms underlying the interaction between ncRNAs and m6A modification in cancer stemness remain incompletely understood. However, several key signaling pathways, such as Wnt/β-catenin, MAPK, Hippo, and JAK/STAT3 pathway, have been implicated in elucidating the underlying interplay mechanisms between m6A modification and ncRNAs in cancer stemness [159]. In a separate investigation, the lncRNA MALAT1 was identified to suppress AngII-induced ROS generation and VSMC proliferation and migration by promoting the expression of GPX4. This effect can be reversed by siRNA targeting GPX4 [149]. The clinical relevance of ncRNAs and the GSH-GPX4 pathway holds considerable importance across different scenarios, encompassing areas such as cancer stemness and ferroptosis. Additional investigations are warranted to comprehensively grasp the intricate interplay mechanisms linking ncRNAs and the GSH-GPX4 pathway. Moreover, efforts are required to devise tailored therapeutic approaches that leverage these interactions for the management of diverse diseases.

The potential utility of targeting ncRNAs and the GSH-GPX4 pathway across a spectrum of clinical contexts, including cancer, NDs, and CVDs, has been extensively investigated. ncRNAs, such as miRNAs, lncRNAs, and circRNAs, are recognized for their pivotal roles in these pathological conditions. In cancer, ncRNAs have emerged as promising diagnostic and prognostic indicators. For instance, in colorectal cancer, exosomal ncRNAs, encompassing lncRNAs and circRNAs, show promise as potential biomarkers for early detection and prognostication of disease progression [160]. Similarly, in BrC, exosomal ncRNAs modulate target genes and pathways, shedding light on the disease's molecular underpinnings [161]. NDs implicate ncRNAs in the regulation of various pathways. For example, miRNAs interact with the circadian rhythm, which is crucial for reproductive regulation and is disrupted in polycystic ovary syndrome (PCOS), hinting at their potential as prognostic indicators [162]. Additionally, circRNAs regulate gene expression implicated in NDs like Alzheimer's disease [163]. Dysregulation of ncRNAs is implicated in CVD pathogenesis. miRNAs, for instance, modulate CVDs such as hypertension and atherosclerosis. circRNAs also play roles in CVDs like heart failure [163,164]. The GSH-GPX4 pathway, pivotal in oxidative stress regulation, is modulated by ncRNAs. For instance, miRNAs regulate GPX4 expression, an enzyme crucial in the GSH-GPX4 pathway, across diverse diseases, including cancer and NDs [162]. Targeting ncRNAs and the GSH-GPX4 pathway in clinical scenarios holds promise for novel diagnostic, prognostic, and therapeutic avenues. However, further elucidation of their molecular mechanisms and clinical applications is warranted.

## 8 Future directions

Future research in the next five years is poised to witness substantial advancements in comprehending the roles of ncRNAs, the GSH-GPX4 pathway, and ferroptosis across various diseases, paving the way for potential therapeutic interventions. Continual endeavors are focused on the advancement of machine learning methodologies for the classification of ncRNAs. Furthermore, the application of CRISPR/Cas technology in ncRNA, particularly in plant biology, holds promise for yielding deeper insights and practical applications in this domain [[165], [166], [167]].

Emerging research underscores the therapeutic potential of targeting the GSH-GPX4 pathway and ferroptosis in a spectrum of diseases, including spinal cord injury and alcoholic liver disease. The pursuit of anti-ferroptosis drugs and the identification of promising therapeutic targets within this pathway offer prospects for the development of innovative treatment modalities for these disorders [[168], [169], [170], [171]]. The forthcoming years are anticipated to witness an increased emphasis on translating research findings related to ncRNA, the GSH-GPX4 pathway, and ferroptosis into clinical applications. This endeavor may encompass the advancement of diagnostic and treatment interventions grounded in the understanding of these elements' roles in disease pathogenesis. In essence, the forthcoming half-decade is poised to mark significant strides in unraveling the intricacies of ncRNA, the GSH-GPX4 pathway, and ferroptosis, with a particular spotlight on their contributions to disease processes and the prospective clinical applications thereof.

## 9 Conclusion

This overview comprehensively explores the multifaceted functions of ncRNAs in orchestrating the GSH-GPX4 pathway of ferroptosis. By delving into the intricate regulatory mechanisms underlying ferroptosis induction and modulation, this review highlights the critical involvement of ncRNAs in fine-tuning key components of the GPX4 pathway. The elucidation of specific ncRNAs, including circRNAs, lncRNAs, and miRNAs targeting crucial elements of the GSH-GPX4 axis, underscores their possibilities as targets for treatment for illnesses that involve dysregulated ferroptosis. Furthermore, this review underscores the therapeutic potential of targeting ncRNAs to overcome DR in cancerous cells, thereby enhancing treatment efficacy and improving patient outcomes. Overall, by shedding light on the regulatory functions of ncRNAs within the GPX4 pathway, this evaluation offers a solid foundation for further studies and therapeutic advancements in the realm of PCD and disease management.

## Data availability

No data was used for the research described in the article.

## CRediT authorship contribution statement

**Sadique Hussain:** Formal analysis, Data curation. **Gaurav Gupta:** Investigation, Formal analysis. **Moyad Shahwan:** Methodology, Investigation. **Pooja Bansal:** Project administration, Methodology. **Harpreet Kaur:** Validation, Supervision. **Mahamedha Deorari:** Project administration, Methodology. **Kumud Pant:** Project administration, Formal analysis. **Haider Ali:** Project administration, Data curation. **Sachin Kumar Singh:** Methodology, Investigation. **Venkata Sita Rama Raju Allam:** Project administration, Formal analysis. **Keshav Raj Paudel:** Project administration, Methodology. **Kamal Dua:** Writing – original draft, Methodology. **Vinoth Kumarasamy:** Validation, Software. **Vetriselvan Subramaniyan:** Data curation, Conceptualization.

## Declaration of competing interest

The authors declare that they have no known competing financial interests or personal relationships that could have appeared to influence the work reported in this paper.
